# Combination interleukin-23p19 inhibitor and Janus kinase inhibitor rescue for steroid-refractory acute severe ulcerative colitis in pregnancy complicated by primary sclerosing cholangitis

**DOI:** 10.1093/gastro/goag095

**Published:** 2026-09-11

**Authors:** Mohammed Nabil Quraishi, Nadeen Omar, Hatim Taha, Sherina AlShamshi, Menatullah Fadda, Zaher Koutoubi

**Affiliations:** Department of Gastroenterology, Sheikh Shakhbout Medical City, PureHealth, Abu Dhabi, United Arab Emirates; Institute of Cancer and Genomic Sciences, University of Birmingham, Birmingham, United Kingdom; College of Medicine and Health Sciences, Khalifa University of Science and Technology, Abu Dhabi, United Arab Emirates; Department of Gastroenterology, Sheikh Shakhbout Medical City, PureHealth, Abu Dhabi, United Arab Emirates; Department of Gastroenterology, Sheikh Shakhbout Medical City, PureHealth, Abu Dhabi, United Arab Emirates; Patient co-author, Abu Dhabi, United Arab Emirates; Department of Obstetrics and Gynaecology, Sheikh Shakhbout Medical City, PureHealth, Abu Dhabi, United Arab Emirates; Digestive Disease Institute, Cleveland Clinic Abu Dhabi, Abu Dhabi, United Arab Emirates

## Introduction

In pregnancy, active inflammatory bowel disease (IBD), rather than the drugs used to control it, is the principal driver of adverse maternal and foetal outcomes. This situation becomes difficult once the therapies with the best-established gestational safety are exhausted. Anti-tumour necrosis factor agents, thiopurines, vedolizumab and ustekinumab carry reassuring pregnancy data [1], whereas interleukin (IL)-23p19 inhibitors have only limited human pregnancy experience and Janus kinase (JAK) inhibitors are generally avoided despite a growing evidence base in acute severe ulcerative colitis (ASUC) [1, 2]. Coexistent primary sclerosing cholangitis (PSC) marks a phenotype in which the colitis is frequently difficult to control and experience with advanced therapies is limited [3]. We report combined risankizumab and tofacitinib as rescue therapy for steroid-refractory ASUC in pregnancy in a woman with PSC-IBD.

## Case report

A 25-year-old Emirati woman had pancolonic ulcerative colitis (Montreal A1, E3) diagnosed in 2016, with PSC diagnosed in December 2023 on magnetic resonance cholangiopancreatography (right posterior segmental biliary stricture, stable in April 2025), managed with standard-dose ursodeoxycholic acid, without bacterial cholangitis. Infliximab (2021) had been stopped for immunogenic loss of response (anti-drug antibody titre 424), and vedolizumab, which achieved deep endoscopic remission (Mayo endoscopic subscore 0, April 2025), was discontinued in November 2025 after a missed infusion and relapse (Table S1).

She relapsed in the early second trimester, biochemically discordant from the outset: at 11 weeks (19 October 2025) faecal calprotectin was already 4,619 μg/g while C-reactive protein (CRP) was only 18.5 mg/L. She remained symptomatic despite intravenous then oral corticosteroids, with up to six bloody stools daily on prednisolone 35 mg (partial Mayo 6). Risankizumab was started in November 2025 (intravenous induction 1,200 mg; 360 mg subcutaneously every 8 weeks), delayed by an insurance rejection. At ∼17 weeks (December 2025), she was admitted with acute severe UC (bloody diarrhoea up to 12 times daily, tachycardia); flexible sigmoidoscopy showed an Ulcerative Colitis Endoscopic Index of Severity score of 7 and calprotectin 4,215 μg/g. She received intravenous methylprednisolone and a 4-week course of off-label oral vancomycin (125 mg four times daily) directed at luminal PSC-IBD activity, for which uncontrolled studies suggest benefit [4], without clear response.

With infliximab precluded, vedolizumab failed and ciclosporin unsuitable, colectomy was recommended but declined after counselling about the maternal and foetal risks of uncontrolled disease. Given systemic stability despite severe, steroid-refractory disease, tofacitinib was proposed as short induction cover on the basis of randomized and meta-analytic evidence in ASUC [5], given off-label alongside risankizumab after multidisciplinary review and consent that it is contraindicated in pregnancy with limited safety data. Pneumocystis jirovecii pneumonia prophylaxis and thromboprophylaxis were given during this period.

**Figure 1:**
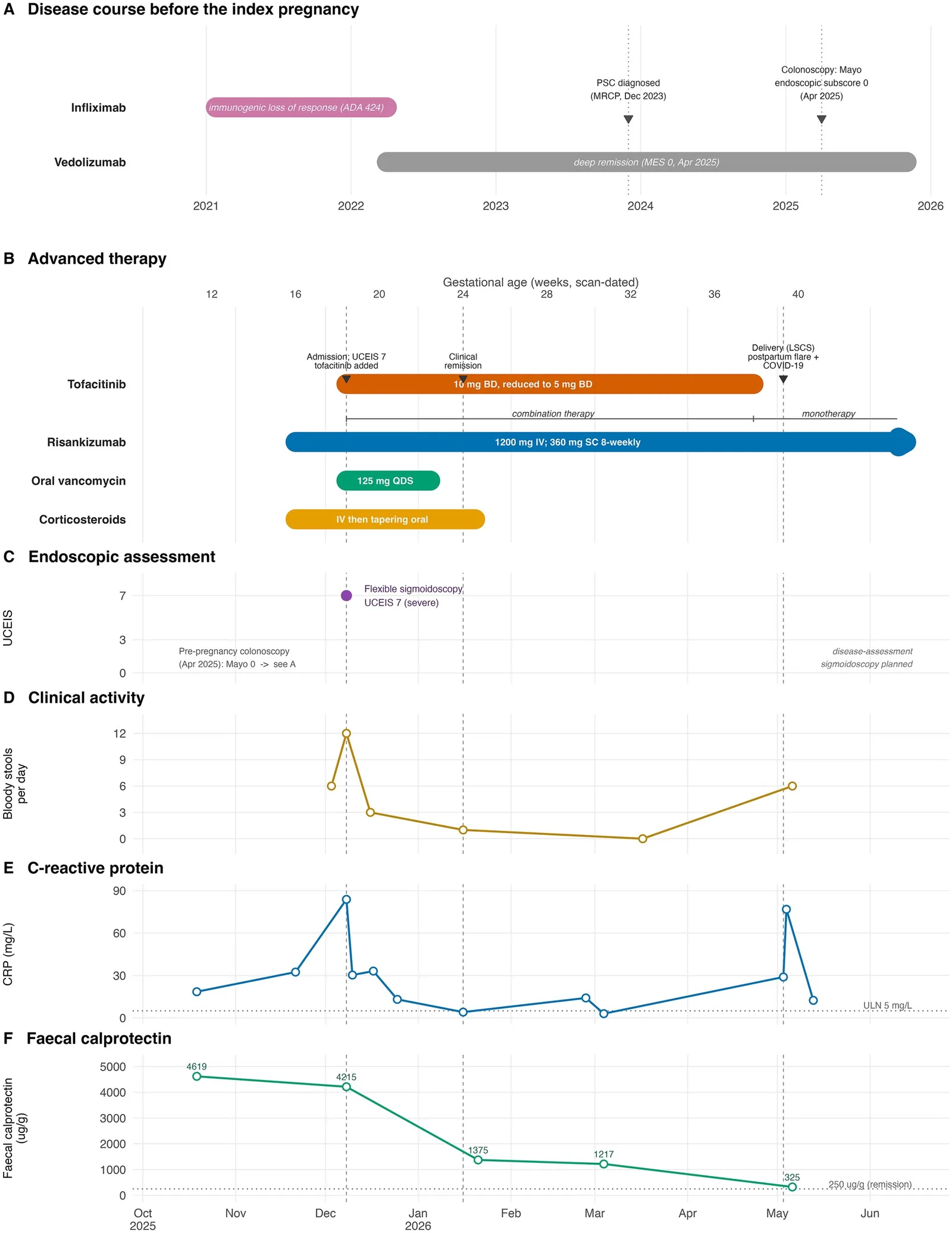
Disease course, therapy and biomarker trajectories. (A) Disease course before the index pregnancy: infliximab (stopped for immunogenic loss of response) and vedolizumab exposure, with primary sclerosing cholangitis diagnosed on MRCP (December 2023) and deep endoscopic remission (Mayo endoscopic subscore 0) at surveillance colonoscopy in April 2025. (B) Advanced-therapy exposure across the index pregnancy and postpartum period, with key clinical events and a scan-dated gestational-age axis; risankizumab was commenced during a steroid-refractory flare (intravenous induction 1,200 mg, then 360 mg subcutaneously every 8 weeks) and, after intravenous corticosteroids failed and colectomy was declined, off-label tofacitinib (10 mg twice daily, later reduced to 5 mg twice daily) was added, achieving clinical response. Tofacitinib was stopped 10 days before delivery; risankizumab was briefly held during postpartum COVID-19 and resumed as monotherapy. Oral vancomycin denotes off-label therapy directed at luminal PSC-IBD activity (no clear response). (C) Endoscopic assessment (UCEIS) and (D) clinical activity (bloody stools per day). (E) CRP and (F) faecal calprotectin trajectories over the same period. Faecal calprotectin was already grossly elevated in the first trimester (4,619 μg/g, 19 October 2025) when CRP was only 18.5 mg/L, remained markedly elevated through pregnancy despite clinical improvement and a normalized CRP, and fell only postpartum, illustrating clinical-biochemical discordance; the dashed line marks the 250 μg/g remission threshold. Gestational age is scan-dated (estimated delivery date 8 May 2026). Abbreviations: ADA, anti-drug antibody; CRP, C-reactive protein; LSCS, lower-segment caesarean section; MES, Mayo endoscopic subscore; MRCP, magnetic resonance cholangiopancreatography; PSC-IBD, primary sclerosing cholangitis-associated inflammatory bowel disease; UCEIS, Ulcerative Colitis Endoscopic Index of Severity; ULN, upper limit of normal.

Tofacitinib 10 mg twice daily was commenced in preference to colectomy; she responded within days (three motions daily, resolving pain, falling CRP) and was discharged. CRP normalized by mid-January 2026 (4.1 mg/L), but faecal calprotectin remained elevated (1,375 μg/g in January; 1,217 μg/g in March). Tofacitinib was reduced to 5 mg twice daily in March 2026 and stopped on 23 April 2026, 10 days before delivery. She delivered a liveborn infant at term (39 weeks and 2 days) by caesarean section for obstetric indications; an isolated hemivertebra was considered unrelated to therapy, and the infant is thriving under orthopaedic follow-up. A self-limiting postpartum flare coincided with COVID-19; risankizumab was briefly held and resumed. She is now in clinical remission on risankizumab monotherapy, with faecal calprotectin 325 μg/g (Fig. 1).

## Discussion and conclusions

Multi-refractory ulcerative colitis, pregnancy and PSC together nearly closed the therapeutic field. Infliximab immunogenicity and vedolizumab failure left no agent combining proven efficacy with established gestational safety. An in-class switch to golimumab was not pursued: recapture after immunogenic failure with high anti-drug antibody titres is unlikely, and golimumab has minimal evidence in acute severe or refractory ulcerative colitis, no intravenous formulation for rescue, and is unavailable on our formulary. Risankizumab was chosen on registry data that have not identified an excess of adverse maternal or neonatal outcomes with IL-23p19 inhibitor exposure [6], though evidence remains insufficient for a firm recommendation; a recent meta-analysis across 17,441 IBD pregnancies derived no pooled estimate for this class [7].

Corticosteroids themselves carry perinatal risk (preterm birth, low birth weight), so continuing them was not neutral [2]. Tofacitinib was added to, not substituted for, risankizumab so that its rapid onset could provide induction cover during the weeks when an IL-23p19 inhibitor takes to act.

This was weighed against the hazards of JAK inhibition. Tofacitinib crosses the placenta and is associated with venous thromboembolism, a concern in the prothrombotic postpartum period; thromboprophylaxis was given. Its human pregnancy dataset, though the largest among JAK inhibitors, largely derives from pregnancies in which the drug was stopped once pregnancy was identified; deliberate continuation remains anecdotal [8]. The same meta-analysis reported a numerically higher rate of early pregnancy loss with JAK inhibitor exposure, but based on only three studies and 33 pregnancies, with very low certainty and likely confounding by indication; it reaffirmed active maternal disease, not drug exposure, as the principal driver of adverse outcomes [7]. Tofacitinib was stopped before delivery given placental transfer and the caution advised during lactation, whereas risankizumab is considered compatible with breastfeeding and continued [9]. The hemivertebra is very unlikely to be treatment-related, as vertebral segmentation completes by approximately the eighth week of gestation, before both the relapse and the introduction of either agent [10].

To our knowledge, combined IL-23p19 and JAK inhibition as rescue for steroid-refractory ASUC in pregnancy has not previously been reported, and extends the now trial-supported use of tofacitinib in ASUC to a population excluded from the pivotal studies. Shared decision-making and prospective registry enrolment are essential. When such patients decline colectomy, controlling severe activity may require options beyond the conventional, within a multidisciplinary, consented framework. This favourable outcome cannot establish the safety or efficacy of either agent or their combination in pregnancy, nor is it a recommendation for their use.

## Supplementary Material

goag095_Supplementary_Data
