# Supplementary material for: Combination interleukin-23p19 inhibitor and Janus kinase inhibitor rescue for steroid-refractory acute severe ulcerative colitis in pregnancy complicated by primary sclerosing cholangitis
Source: Gastroenterol Rep (Oxf). 2026 Sep 11;14:goag095. doi: 10.1093/gastro/goag095 (PMC13568818; doi:10.1093/gastro/goag095)
Supplement: Supplementary file 1 [file goag095_supplementary_data.docx]

**Supplementary Material**

**Supplementary Table 1.** Detailed dated treatment and biomarker timeline for the index case.

| Date | Gestation^a^ | Clinical event/therapy | Investigations/biomarkers |
| --- | --- | --- | --- |
| Apr 2016 | – | Ulcerative colitis diagnosed (elsewhere); pancolitis, Montreal A1, E3 | – |
| Jan 2021 | – | Infliximab commenced | – |
| Apr 2022 | – | Infliximab discontinued (immunogenic secondary loss of response) | Anti-drug antibody titre 424; undetectable trough |
| Apr 2022 | – | Vedolizumab commenced (interrupted in first pregnancy, restarted May 2023) | – |
| Dec 2023 | – | Primary sclerosing cholangitis diagnosed; standard-dose ursodeoxycholic acid commenced | Magnetic resonance cholangiopancreatography: right posterior segmental biliary stricture; cholestatic profile (ALP 180–210 U/L, raised GGT) |
| Apr 2025 | – | Surveillance colonoscopy: deep endoscopic remission on vedolizumab; follow-up biliary imaging stable | Mayo endoscopic subscore 0; histology chronic inactive |
| 19 Oct 2025 | ~11 weeks | First-trimester biomarker baseline (pre-relapse) | Faecal calprotectin 4,619 ug/g; CRP 18.5 mg/L |
| Nov 2025 | ~14 weeks | Relapse after missed vedolizumab infusion; vedolizumab discontinued; intravenous then oral corticosteroids | Up to 6 bloody stools/day on prednisolone 35 mg; partial Mayo 6 |
| Nov 2025 | ~14 weeks | Risankizumab commenced (IV induction 1,200 mg; 360 mg SC every 8 weeks); delayed by insurance rejection | – |
| Dec 2025 | ~17 weeks | Admission with acute severe UC; flexible sigmoidoscopy; intravenous methylprednisolone; four-week oral vancomycin 125 mg four times daily | UCEIS 7; faecal calprotectin 4,215 ug/g; CRP peak 83.8 mg/L |
| Dec 2025 | ~17 weeks | Colectomy recommended and declined; tofacitinib 10 mg twice daily added off-label; PJP prophylaxis and thromboprophylaxis given | Clinical response within days (3 stools/day) |
| Mid-Jan 2026 | – | Corticosteroids weaned; continued dual therapy | CRP normalised (4.1 mg/L); faecal calprotectin 1,375 ug/g (21 Jan) |
| Mar 2026 | – | Tofacitinib de-escalated to 5 mg twice daily | Faecal calprotectin 1,217 ug/g (4 Mar) |
| 23 Apr 2026 | ~38 weeks | Tofacitinib stopped (10 days before delivery) | – |
| 3 May 2026 | 39+2 weeks | Liveborn term infant by caesarean section (obstetric indications); isolated hemivertebra | – |
| May 2026 | Postpartum | Self-limiting flare with COVID-19; brief prednisolone; risankizumab held then resumed 20 May as monotherapy | Faecal calprotectin 325 ug/g (6 May); CRP 76.8 then 12.4 mg/L |
| 1 Jul 2026 | – | Clinical remission on risankizumab monotherapy; surveillance sigmoidoscopy planned | Simple Clinical Colitis Activity Index 1; CRP 11.2 mg/L |

ALP, alkaline phosphatase; CRP, C-reactive protein; GGT, gamma-glutamyl transferase; PJP, Pneumocystis jirovecii pneumonia; UC, ulcerative colitis; UCEIS, Ulcerative Colitis Endoscopic Index of Severity.

^a^Gestational ages are scan-dated and approximate.
